# Impact of Pathologic Complete Response on the Prognosis of Triple-Negative Breast Cancer Patients: A Cohort Study

**DOI:** 10.7759/cureus.37396

**Published:** 2023-04-10

**Authors:** Rafael Everton Assunção Ribeiro da Costa, Fergus Tomás Rocha de Oliveira, Ana Lúcia Nascimento Araújo, Sabas Carlos Vieira

**Affiliations:** 1 Health Science Center, State University of Piauí, Teresina, BRA; 2 Tocogynecology, Oncocenter, Teresina, BRA

**Keywords:** pathologic complete response, prognosis, neoadjuvant chemoradiotherapy, hr-/pr-/her2- breast cancer, survival analysis

## Abstract

Introduction

Triple-negative breast cancer (TNBC) is a molecular subtype in which estrogen (ER)/progesterone receptor (PR) and human epidermal growth receptor 2 (HER2) expression does not occur. The objective of this study was to analyze the impact of pathologic complete response (pCR) after neoadjuvant chemotherapy on the prognosis of triple-negative breast cancer (TNBC) patients.

Methods

This cohort study was conducted in a private-sector oncology clinic located in the city of Teresina, Brazil. Medical charts of 532 breast cancer patients treated from 2007 to 2020 were analyzed. Of these patients, 83 women with TNBC were selected (10 patients were excluded from the study). Univariate and multivariate analyses (Cox regression) were performed to evaluate the impact on patient survival, comparing patients with or without pCR. A significance level of 5% was set. Overall survival (OS) and disease-free survival (DFS) curves were constructed according to the Kaplan-Meier model.

Results

Angiolymphatic invasion and positive sentinel lymph node were associated with a lower OS and/or DFS in TNBC (p<0.05). The 10-year OS was 78% and 49%, and the 10-year DFS was 97% and 32% in patients with or without pCR, respectively.

Conclusion

pCR after neoadjuvant chemotherapy was associated with improvement in OS and DFS in TNBC patients.

## Introduction

Breast cancer (BC) is the most frequently diagnosed female cancer, accounting for 23% of the total cancer cases. It is also considered a molecularly diverse disease. Breast cancer is classically divided into three types: (a) estrogen receptor (ER) or progesterone receptor (PR)-positive; (b) human epidermal growth factor receptor 2 (HER2)-positive (erbB2 amplification) with or without ER/PR positivity; (c) triple-negative breast cancer (TNBC), which is defined by the lack of ER/PR expression and HER2 amplification. Immunohistochemistry and DNA microarray technology (cDNA) have identified five molecular subtypes of BC: 1) luminal A (ER-positive and/or PR-positive and HER2-negative); 2) luminal B (ER-positive and/or PR-positive and HER2-positive); 3) HER2 overexpression (ER and PR-negative and HER2-positive); 4) basal-like (ER/PR/HER2-negative, cytokeratin 5/6-positive and/or epidermal growth factor receptor-positive); 5) normal breast like (tumors that are not included in any of the previous categories) [1,2].

TNBC accounts for about 10-15% of BC. TNBC is more common in younger (around age 53) and obese women. It has a higher prevalence in premenopausal black and Hispanic women. Furthermore, there is also a correlation between the occurrence of TNBC and pathogenic variants in breast cancer gene 1 (BRCA1) and breast cancer gene 2 (BRCA2) in more than 20% of TNBC patients. Regarding the molecular subtype, 70% of TNBC are basal-like (the majority of basal-like BC are also TNBC). In a recent genomic analysis of TNBC, four subtypes were confirmed: (I) luminal androgen receptor; (II) mesenchymal; (III) basal-like immunosuppressed; (IV) basal-like immunoactivated. The basal-like immunoactivated subtype is associated with a better prognosis, indicating a better outcome in TNBC with lymphocytic infiltration [3].

TNBC has a more aggressive course, and the patient mortality rate is around 40% in the first five years after diagnosis. The recurrence rate after surgery is about 25%, and the mean survival time after the occurrence of metastasis is only 13.3 months. After tumor recurrence, the mortality rate is 75% in TNBC patients. In TNBC, the sites most frequently affected by metastasis are the brain and visceral organs. Neoadjuvant chemotherapy is essentially administered for many years of systemic treatment in TNBC since its molecular phenotype makes it less sensitive to endocrine therapy. Molecular targeted therapy and adjuvant chemotherapy are also less effective in these tumors [4]. Nevertheless, in the current scenario, three targeted therapies became available and have been approved for systemic treatment: polymerase (PARP) inhibitors - olaparib and talazoparib in patients with BRCA1 and BRCA2 gene mutation and atezolizumab combined with nab-paclitaxel in patients with advanced TNBC and programmed death-ligand 1 (PD-L1)+ [5].

The occurrence of pathologic complete response (pCR, absence of tumor and lymph node involvement) after neoadjuvant chemotherapy in BC patients is associated with better long-term outcomes, with longer overall survival (OS) and disease-free survival (DFS), in addition to a lower risk of death and tumor recurrence. Although TNBC tumors have a more aggressive natural history, they are more chemoresponsive than non-TNBC tumors, especially concerning neoadjuvant chemotherapy. TNBC tumors also have higher pCR rates [6,7]. In the last years, the use of adjuvant capecitabine has improved the prognosis of patients with HER2-negative tumors that have residual disease after neoadjuvant chemotherapy non-complete response (non-pCR). Capecitabine has been shown to increase OS and DFS in these cases [8].

The objective of this study was to analyze the impact of pathologic complete response (pCR) after neoadjuvant chemotherapy on the prognosis of TNBC patients.

## Materials and methods

This is a retrospective cohort study. In a private sector oncology clinic located in the city of Teresina (PI), Brazil, medical records of 532 breast cancer patients treated from 2007 to 2020 were evaluated. Of the total number of records, 93 (17%) were of women diagnosed with TNBC - a negative expression of ER/PR (less than 10%) by immunohistochemistry (IHC) and HER2 (either by IHC 0-1 or by fluorescent in situ hybridization (FISH)-negative if 2+ on IHC) [2]. Ten medical records were excluded (four patients were still under treatment, and six records had incomplete or missing data). Therefore, the final sample included 83 women diagnosed with TNBC.

Data was tabulated by using Microsoft Excel 2010 program (Microsoft® Corp., Redmond, USA). Statistical analysis was carried out in the Stata 14 program (StataCorp LLC, College Station, USA), USA. Absolute (n) and relative (%) frequencies of all variables adopted (age at diagnosis, clinical stage, grade of differentiation, angiolymphatic invasion, sentinel lymph node status, Ki-67 [cut-off at 60%], and recurrence) were calculated for descriptive analysis (log-rank test). The prognostic value of Ki-67 in TNBC remains controversial, partly due to the lack of agreement on the cut-off point, ranging from 10 to 61%. Based on the analysis conducted by Zenzola et al., the cut-off point of 60% was used in this study [9]. On univariate and multivariate (Cox regression) analyses, the prognostic factors related to a reduction in OS and/or DFS in study patients were evaluated. Patients were divided into two groups: 1) pCR - considered in cases of absence of tumor and lymph node involvement after the neoadjuvant chemotherapy; and 2) non-pCR [6]. For univariate analysis, the hazard ratios with their respective 95% confidence intervals (CI) were estimated. Values of p<0.05 were considered significant. A stepwise Cox proportional hazards regression model was used to compare the chances of not occurring death and recurrence among pCR and non-pCR patients throughout the follow-up period of the study - a multivariate analysis. OS and DFS curves were constructed according to the Kaplan-Meier model.

Follow-up of these oncology patients started at the TNBC diagnosis time by biopsy, histopathology, and IHC (with or without FISH). In the OS and DFS analyses, the occurrence of death and cancer recurrence was considered as failure, respectively. The survival analysis time started in November 2007, and the censoring time was December 2020 (a period of 157 months).

## Results

Table 1 shows the distribution of live patients among the pCR and non-pCR tumors. Among non-pCR patients, angiolymphatic invasion and recurrence were the only variables associated with higher mortality (p < 0.05). No variable was related to a significant increase in mortality among pCR patients.

**Table 1:** Distribution of live patients *The patient had been classified as clinical stage IV but was assigned III-C. **The patient had been classified as having a grade 1 (G1) tumor (grade of differentiation) but she was allocated to the grade 2 (G2) group. pCR - pathologic complete response; non-pCR - non-complete response

Variables	Non-pCR		p-value (log-rank test)	pCR		p-value (log-rank test)
	Total n (%)	Live patients n (%)		Total n (%)	Live patients n (%)	
Age at diagnosis (years)						
Up to 59	24 (58.5)	17 (70.8)	0.397	26 (61.9)	26 (100.0)	0.065
60 or older	17 (41.5)	14 (82.4)		16 (38.1)	14 (87.5)	
Clinical stage						
I-B	7 (17.1)	6 (85.7)	0.26	18 (42.9)	18 (100.0)	0.154
II-B	12 (29.2)	11 (91.7)		12 (28.6)	11 (91.7)	
III-B	15 (36.6)	10 (66.7)		8 (19.0)	8 (100.0)	
III-C^*^	7 (17.1)	4 (57.1)		4 (9.5)	3 (75.0)	
Grade of differentiation						
G2^**^	24 (58.5)	18 (75.0)	0.914	21 (50.0)	21 (100.0)	0.147
G3	17 (41.5)	13 (76.5)		21 (50.0)	19 (90.5)	
Lymphatic invasion						
No	31 (75.6)	29 (93.6)	<0.001	32 (76.2)	31 (96.9)	0.373
Yes	10 (24.4)	2 (20.0)		10 (23.8)	9 (90.0)	
Vascular invasion						
No	34 (82.9)	30 (88.2)	<0.001	33 (78.6)	32 (97.0)	0.313
Yes	7 (17.1)	1 (14.3)		9 (21.4)	8 (88.9)	
Sentinel lymph node status						
Negative	28 (68.3)	23 (82.1)	0.153	35 (83.3)	34 (97.1)	0.195
Positive	13 (31.7)	8 (61.5)		7 (16.7)	6 (85.7)	
Ki-67						
≤ 60	18 (43.9)	16 (88.9)	0.08	21 (50.0)	19 (90.5)	0.147
> 60	23 (56.1)	15 (65.2)		21 (50.0)	21 (100.0)	
Recurrence						
No	22 (53.7)	21 (95.4)	0.001	40 (95.2)	38 (95.0)	0.746
Yes	19 (46.3)	10 (52.6)		2 (4.8)	2 (100.0)	

The univariate analysis (Table 2) showed that only angiolymphatic invasion was a prognostic factor for a decrease in OS among non-pCR patients (p <0.05). This analysis showed no prognostic factors for OS reduction among pCR patients.

**Table 2:** Univariate analysis of overall survival in study patients *The patient had been classified as clinical stage IV but was assigned III-C. **The patient had been classified as a grade 1 (G1) tumor (grade of differentiation) but was allocated to the grade 2 (G2) group. pCR - pathologic complete response; non-pCR - non-complete response; HR - hazard ratios; CI - confidence interval

Variables	Non-pCR				p-value (Wald's test)		pCR						p-value (Wald's test)	
	n (%)	Deaths n (%)		HR (95% CI)			n (%)		Deaths n (%)		HR (95% CI)			
Age at diagnosis (years)														
Up to 59	24 (58.5)	7 (29.2)		1			2 (61.9)		-		-			
60 and over	17 (41.5)	3 (17.6)		0.71 (0.18-2.83)	0.624		16 (38.1)		2 (12.5)		-		-	
Clinical stage														
I-B	7 (17.1)	1 (14.3)		1			18 (42.9)		-		-			
II-B	12 (29.2)	1 (8.3)		0.38 (0.02-6.02)	0.489		12 (28.6)		1 (8.3)		-		-	
III-B	15 (36.6)	5 (33.3)		3.06 (0.35-26.46)	0.309		8 (19.0)		-		-		-	
III-C^*^	7 (17.1)	3 (42.9)		4.26 (0.42-43.35)	0.221		4 (9.5)		1 (25.0)		-		-	
Grade of differentiation														
G2^**^	24 (58.5)	6 (25.0)		1			21 (50.0)		-		-			
G3	17 (41.5)	4 (23.5)		0.72 (0.20-2.56)	0.612		21 (50.0)		2 (9.5)		-		-	
Lymphatic invasion														
No	31 (75.6)	2 (6.4)		1			32 (76.2)		1 (3.1)		-			
Yes	10 (24.4)	8 (80.0)		37.84 (4.57-313.09)	0.001		10 (23.8)		1 (10.0)		-		-	
Vascular invasion														
No	34 (82.9)	4 (11.8)		1			33 (78.6)		1 (3.0)		-			
Yes	7 (17.1)	6 (85.7)		9.75 (2.37-40.12)	0.002		9 (21.4)		1 (11.1)		-		-	
Sentinel lymph node status														
Negative	28 (68.3)	5 (17.9)		1			35 (83.3)		1 (2.9)		-			
Positive	13 (31.7)	5 (38.5)		2.58 (0.74-9.02)	0.136		7 (16.7)		1 (14.3)		-		-	
Ki-67														
≤ 60	18 (43.9)	2 (11.1)		1			21 (50.0)		2 (9.5)		-			
> 60	23 (56.1)	8 (34.8)		4.13 (0.87-19.54)	0.073		21 (50.0)		-		-		-	
Recurrence														
No	22 (53.7)	1 (4.6)		1			40 (95.2)		2 (5.0)		-			
Yes	19 (46.3)	9 (47.4)		7.34 (0.93-58.05)	0.059		2 (4.8)		-		-		-	

The distribution of patients without recurrence among the pCR and non-pCR tumors (Table 3) demonstrated that lymphatic invasion and positive sentinel lymph node were the only variables related to a significant increase in recurrence among non-pCR patients (p<0.05). No variable was related to a significant increase in recurrence among pCR patients.

**Table 3:** Distribution of patients without recurrence *The patient had been classified as clinical stage IV but was assigned III-C. **The patient had been classified as a grade 1 (G1) tumor (grade of differentiation) but was allocated to the grade 2 (G2) group. pCR - pathologic complete response; non-pCR - non-complete response

Variables	Non-pCR		p-value (log-rank test)	pCR		p-value (log-rank test)
	Total n (%)	Patients without recurrence n (%)		Total n (%)	Patients without recurrence N (%)	
Age at diagnosis (years)						
Up to 59	24 (58.5)	13 (54.2)	0.675	26 (61.9)	25 (96.2)	0.414
60 and older	17 (41.5)	11 (64.7)		16 (38.1)	16 (100.0)	
Clinical stage						
I-B	7 (17.1)	6 (85.7)	0.066	18 (42.9)	18 (100.0)	0.506
II-B	12 (29.2)	7 (58.3)		12 (28.6)	11 (91.7)	
III-B	15 (36.6)	9 (60.0)		8 (19.0)	8 (100.0)	
III-C^*^	7 (17.1)	2 (28.6)		4 (9.5)	4 (100.0)	
Grade of differentiation						
G2^**^	24 (58.5)	15 (62.5)	0.853	21 (50.0)	20 (95.2)	0.317
G3	17 (41.5)	9 (52.9)		21 (50.0)	21 (100.0)	
Lymphatic invasion						
No	31 (75.6)	21 (67.7)	0.011	32 (76.2)	31 (96.9)	0.563
Yes	10 (24.4)	3 (30.0)		10 (23.8)	10 100.0)	
Vascular invasion						
No	34 (82.9)	22 (64.7)	0.155	33 (78.6)	32 (97.0)	0.59
Yes	7 (17.1)	2 (28.6)		9 (21.4)	9 (100.0)	
Sentinel lymph node status						
Negative	28 (68.3)	19 (67.9)	0.025	35 (83.3)	34 (97.1)	0.674
Positive	13 (31.7)	5 (38.5)		7 (16.7)	7 (100.0)	
Ki-67						
≤ 60	18 (43.9)	13 (72.2)	0.066	21 (50.0)	21 (100.0)	0.317
> 60	23 (56.1)	11 (47.8)		21 (50.0)	20 (95.2)	

The univariate analysis (Table 4) showed that lymphatic invasion and positive sentinel lymph were prognostic factors for reduction in DFS among non-pCR patients (p<0.05). In this analysis, there were no prognostic factors for DFS reduction among pCR patients.

**Table 4:** Univariate analysis of disease-free survival in study patients *The patient had been classified as clinical stage IV but was assigned III-C. **The patient had been classified as a grade 1 (G1) tumor (grade of differentiation) but was allocated to the group 2 (G2) group. pCR - pathologic complete response; non-pCR - non-complete response; HR - hazard ratio; CI - confidence interval

Variables	Non-pCR			p-value (Wald's test)	pCR			p-value (Wald's test)
	n (%)	Recurrences n (%)	HR (95% CI)		n (%)	Recurrences n (%)	HR (95% CI)	
Age at diagnosis (years)								
Up to 59	24 (58.5)	11 (45.8)	1		26 (61.9)	1 (3.8)	-	
60 and older	17 (41.5)	6 (35.3)	0.81 (0.30-2.20)	0.679	16 (38.1)	-	-	-
Clinical stage								
I-B	7 (17.1)	1 (14.3)	1		18 (42.9)	-	-	
II-B	12 (29.2)	5 (41.7)	2.21 (0.26-18.98)	0.469	12 (28.6)	1 (8.3)	-	-
III-B	15 (36.6)	6 (40.0)	3.99 (0.48-33.42)	0.202	8 (19.0)	-	-	-
III-C^*^	7 (17.1)	5 (71.4)	8.12 (0.94-70.30)	0.057	4 (9.5)	-	-	-
Grade of differentiation								
G2^**^	24 (58.5)	9 (37.5)	1		21 (50.0)	1 (4.8)	-	
G3	17 (41.5)	8 (47.1)	0.91 (0.35-2.40)	0.855	21 (50.0)	-	-	-
Lymphatic invasion								
No	31 (75.6)	10 (32.3)	1		32 (76.2)	1 (3.1)	-	
Yes	10 (24.4)	7 (70.0)	3.43 (1.23-9.55)	0.018	10 (23.8)	-	-	-
Vascular invasion								
No	34 (82.9)	12 (35.3)	1		33 (78.6)	1 (3.0)	-	
Yes	7 (17.1)	5 (71.4)	2.13 (0.72-6.29)	0.17	9 (21.4)	-	-	-
Sentinel lymph node status								
Negative	28 (68.3)	9 (32.1)	1		35 (83.3)	1 (2.9)	-	
Positive	13 (31.7)	8 (61.5)	2.82 (1.08-7.37)	0.034	7 (16.7)	-	-	-
Ki-67								
≤ 60	18 (43.9)	5 (27.8)	1		21 (50.0)	-	-	
> 60	23 (56.1)	12 (52.2)	2.55 (0.90-7.28)	0.079	21 (50.0)	1 (4.8)	-	-

**Figure 1:**
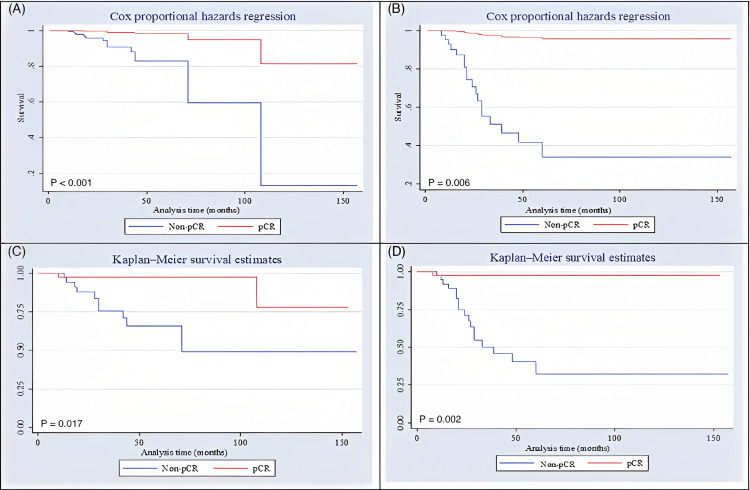
Curves of study patients (A): chances of not occurring death; (B): chances of not occurring tumor recurrence; (C): overall survival; (D): disease-free survival pCR - pathologic complete response (42 patients); non-pCR - non-complete response (41 patients)

Survival curves proportional to the risk of death and recurrence (Figure 1A and 1B, respectively) of study patients showed a higher probability of not occurring death and recurrence in pCR patients than in non-pCR patients during the whole follow-up study period (p<0.05). Regarding OS and DFS, the survival curves (Figure 1C and 1D, respectively) showed that the 10-year OS was 49% in non-pCR and 78% in pCR (p=0.017). The 10-year DFS was 32% in non-pCR and 97% in pCR (p=0.002).

## Discussion

In this study, 42 patients (50.6%) achieved pCR after neoadjuvant chemotherapy. It has been well-established that pCR rates are higher in TNBC than in non-triple negative tumors [10,11]. Wu et al. conducted a meta-analysis to assess pCR in TNBC, finding pCR rates of 28.9% in TNBC tumors and 12.5% in non-TNBC tumors [12]. However, TNBC is a heterogeneous disease, divided into six subtypes: a) two basal-like (BL1 and BL2); b) one immunomodulatory (IM); c) one mesenchymal (M); d) one mesenchymal stem-like (MSL); e) one luminal androgen receptor (LAR). BL1 subtype is associated with higher pCR rates [13,14]. Santonja et al. analyzed 125 patients with TNBC tumors and also determined pCR rates in the six subtypes of TNBC, showing that the highest pCR rate was seen in the BL1 subtype (47.1%) and the lowest in the LAR subtype (14.3%). Furthermore, pCR also depends on the chemotherapy regimen adopted since some breast cancer subtypes are more chemosensitive or chemoresistant to certain treatment regimens [13,15]. In the current study, this classification could not be applied since the genetic sequencing of the presented tumors was not carried out.

The angiolymphatic invasion was associated with a reduction in OS among TNBC patients who did not achieve pCR. Lymphatic invasion and positive sentinel lymph node were related to a lower DFS. Therefore, the lymphatic invasion was the only prognostic factor solely related to a decrease in OS and DFS in TNBC patients who did not attain pCR.

A study by Asaga et al. analyzed the prognostic factors (also evaluating the impact of pCR and non-pCR) in the reduction of OS and DFS in 135 TNBC patients undergoing neoadjuvant chemotherapy, showing that pCR is highly related to improvement in OS and DFS, which may explain why analyses in this study did not find any isolated prognostic factor related to a worse OS and DFS in TNBC patients with pCR [16]. Shao et al. also conducted an analysis similar to that of Asaga et al. with 53 TNBC patients after neoadjuvant chemotherapy, showing that lymphatic invasion is also a prognostic factor for decreased OS and DFS in these patients [17].

Studies found in the literature indicate higher OS and DFS in TNBC and pCR patients in comparison to non-pCR patients [18-20]. A retrospective cohort of 2007 patients conducted by De-la-Cruz-Ku et al. showed that the 10-year OS and DFS were 47% and 41% in TNBC, respectively [21]. Nevertheless, due to the heterogeneity of TNBC, OS, and DFS values are greatly dependent on sample characteristics, such as mean age at diagnosis, clinical stage, grade of differentiation, angiolymphatic invasion, sentinel lymph node status, Ki-67, and recurrence, among others [22]. For example, the study developed by Fayaz et al. with 353 TNBC patients showed that 10-year OS and DFS were 66% and 59%, respectively [23]. In this study, patients achieving pCR had 10-year OS (78%) and DFS (97%) rates that were higher than rates found in TNBC without pCR (49% and 32%, respectively) and TNBC in general. In addition, the probability of not occurring death and tumor recurrence was higher during the entire follow-up period of the study. Therefore, in agreement with the results found by Asaga et al. and Shao et al. [16,17], the lack of pCR was also shown to be an important prognostic factor in decreasing OS and DFS and increasing the risk of death and tumor recurrence in this study of TNBC.

Limitations of this study include limited sample size, its retrospective nature, and performance in a single health center.

## Conclusions

Among non-pCR patients, only angiolymphatic invasion was shown to be a prognostic factor for a significative decrease in OS. There were no prognostic factors for OS reduction among pCR patients. Lymphatic invasion and positive sentinel lymph node were shown to be prognostic factors in the reduction of DFS among non-pCR patients (p<0.05). There were no prognostic factors for DFS reduction among pCR patients. Survival curves proportional to the risk of death and recurrence showed a higher probability of not occurring death and recurrence in pCR patients than in non-pCR patients during the whole follow-up study period (p<0.05). Regarding OS and DFS, the 10-year OS was 49% in non-pCR and 78% in pCR (p=0.017). The 10-year DFS was 32% in non-pCR and 97% in pCR (p=0.002). In this way, it is possible to conclude that pathologic complete response following neoadjuvant chemotherapy was associated with better overall and disease-free survival in triple-negative breast cancer patients.
